# Dangers of Kerosene

**Published:** 1871-04

**Authors:** 


### DANGERS OF KEROSENE.

Kerosene oil can be made which will not explode, and any one with two ideas can find out whether it is fit to be used in a lamp or not. Take a saucer of warm water, say a hundred degrees, that is not hotter than a healthy heat, pour some of the oil on the water, and apply a flame to it; if the flame sets it on fire, it is not safe; if it does not set it on fire, it is safe, and you should tell every friend and neighbor where they can purchase kerosene oil which cannot blow them up, burn them to death, or scare them out of their wits. If the clothing should take fire, lie down instantly and roll yourself up in a carpet, blanket, or anything else at hand. If you stand up while the clothes are on fire, the flame rises about the head and face, and breathing it in is certain death. If you lie down, the flame goes upward, and the face is untouched. Rolling yourself in a carpet or blanket smothers the fire.

